# LZTR1 is a melanoma oncogene that promotes invasion and suppresses apoptosis

**DOI:** 10.1038/s41388-025-03538-2

**Published:** 2025-08-30

**Authors:** Antonella Bacchiocchi, Michael Mak, Zerin Mahzabin Khan, Xiangyu Gong, Mario Sznol, Zhenkun Na, Haomiao Su, Lok Hei Chan, Qin Yan, Dejian Zhao, Ryland D. Mortlock, James Knight, Sarah A. Slavoff, Ruth Halaban

**Affiliations:** 1 Department of Dermatology, Yale University School of Medicine, New Haven, CT USA; 2 Yale Cancer Center, Yale School of Medicine, New Haven, CT USA; 3 Department of Biomedical Engineering, Yale University, New Haven, CT USA; 4 Department of Pharmacological Sciences, Stony Brook University, Stony Brook, NY USA; 5 Harvard John A. Paulson School of Engineering and Applied Sciences, Harvard University, Boston, MA USA; 6 Wyss Institute for Biologically Inspired Engineering, Harvard University, Boston, MA USA; 7 Department of Medicine, Yale Cancer Center, Yale University School of Medicine, New Haven, CT USA; 8 Department of Chemistry, Yale University, New Haven, CT USA; 9 Hangzhou Institute of Medicine, Chinese Academy of Sciences, Hangzhou, China; 10 Department of Pathology, Yale University School of Medicine, New Haven, CT USA; 11 Yale Center for Genome Analysis, Yale University, New Haven, CT USA; 12 Department of Molecular Biophysics and Biochemistry, Yale University, New Haven, CT USA; 13 Institute for Biomolecular Design and Discovery, Yale University, West Haven, CT USA

**Keywords:** Oncogenes, Prognostic markers

## Abstract

Leucine zipper like transcription regulator 1 (LZTR1) is amplified in acral melanomas, is required for melanocytes and melanoma cell proliferation, and it induces anchorage-independent growth, by yet unknown mechanisms. We therefore performed comprehensive studies to identify its activity in melanomas employing proximity biotinylation and co-immunoprecipitation combined with LC-MS/MS proteomics and molecular characterization. The results show that LZTR1 regulates the ubiquitin proteasome system in melanoma cells and also associates with actin-related proteins and actin cytoskeleton organization. Its downregulation suppresses the protective effect of the autophagy-initiating ULK1 and AMBRA1, regulators of normal cell survival and proliferation, and upregulates the sequestosome 1 (SQSTM1/p62), an autophagic cargo adapter which mediates selective degradation of ubiquitinated proteins. In contrast, overexpression of LZTR1 provides growth advantage under environmental stress, enhancing cell invasion, by activating ERBB3 receptor and its downstream targets PYK2 and SRC tyrosine kinases that regulate the cytoskeleton, actin organization, cell spreading, cell migration and adhesion. LZTR1 is a “safeguard” for melanoma cells under stress and its downregulation can be exploited for melanoma therapy.

## Introduction

Leucine zipper like transcription regulator 1 (LZTR1) is a member of the BTB-Kelch superfamily that is a cullin 3 (CUL3) adaptor protein in complex with E3 ubiquitin-protein ligase [1, 2]. The function of LZTR1 is currently controversial. It is defined a tumor suppressor because it induces the ubiquitination and degradation of RAS family of proteins [2–5], though these results were not confirmed by later studies [6, 7]. We recently showed late-arising amplification of specific genes, including LZTR1, in 38% of acral melanomas (that appear on the palms of the hands, the soles of the feet, or under the nails), that were associated with poor outcome and regional metastasis [8]. Recently, LZTR1 amplification was reported also in 45.5% of East Asian acral melanomas [9]. We demonstrated that LZTR1 is required for melanoma cell proliferation regardless of endogenous levels of expression, because its deletion causes growth arrest and cell death without any effect on RAS [8]. Furthermore, overexpression of LZTR1 in normal human melanocytes confers properties consistent with malignant transformation and initiation of metastasis, such as enhancing anchorage-independent growth, and the formation of three-dimensional clusters in 2D and 3D collagen cultures, reminiscent of a malignant cell phenotype [8]. While the molecular mechanism for these changes remained unknown, we hypothesized that in melanoma, LZTR1 may regulate the stability of target proteins involved in these processes.

In the current study, we performed a comprehensive analysis of LZTR1 functions in several melanoma cell lines derived from acral or sun-exposed tumors bearing B-Raf proto-oncogene (BRAF), GTPase NRAS proto-oncogene (NRAS) or other oncogenic mutations and compared the results to non-melanoma cells. We identified proteins associated with LZTR1 and confirmed that LZTR1 is part of the ubiquitination process in the Golgi complex [1]. We performed proteomic and functional analyses and extended the current information about LZTR1 partners. We show that LZTR1 protects the proteosome machinery, its absence induces apoptosis due to down-regulation of the autophagy-initiating, serine/threonine-protein kinase Unc-51 like kinase-1 (ULK1) and the proteasome regulator autophagy and beclin 1 regulator 1 (AMBRA1), and upregulation of SQSTM1/p62, a protein tightly involved in the autophagic and ubiquitin-proteasome systems. Overexpression of LZTR1 provides growth advantage under environmental stress, enhancing cell invasion, by upregulating and activating ERBB3, the protein tyrosine kinases PYK2/PTK2B and the proto-oncogene, tyrosine kinase SRC, that enhance cell migration and spreading [10–12].

## Materials and methods

### Cell lines

We used melanoma cells derived from acral (YUCRATE, YUHIMO, YUSEEP) or sun-exposed (YUGASP, YUHEF, YUSIK, YUZEST, YUSIV) tumors obtained from the Specimen Resource Core of the Yale SPORE in Skin Cancer [8], non-melanoma cells HEK293 (ATCC catalog number PTA-4488), U-87MG (ATCC catalog number HTB-14), and SCC25 (ATCC catalog number CRL-1628) (Supplementary Table S1). The melanoma cells were grown in OptiMEM medium (Invitrogen, Carlsbad, CA) supplemented with 5% fetal calf serum and antibiotics (Supplementary Table S1). The normal human melanocytes (NBMEL) were freshly isolated from newborn foreskins and grown in medium supplemented with bFGF, IBMX and dbcAMP as described [13]. The Yale melanoma cell lines were characterized by next-generation sequencing [14–16] partially described in Supplementary Table S1.

### Short hairpin RNA (shRNA), Western blotting and cell viability tests

The effects of downregulating target proteins on cell proliferation and signal transduction were tested using puromycin-bearing MISSION lentiviral vectors pLKO.1 shRNA, provided by the YCC Functional Genomics Core at Yale, Dr. David Calderwood and Dr. Ben Turk, Directors.

We chose predesigned shRNA from Sigma (https://www.sigmaaldrich.com/US/en/semi-configurators/shrna?activeLink=selectClones) listed in Supplementary Table 5. Non-Mammalian shRNA SCH002 pLKO.1 vector was used as a negative control [8]. Invitrogen™ ViraPower™ Lentiviral Packaging Mix (catalog no. K497500) was used for lentivirus packaging followed by transfection into HEK293T cells (ATCC catalog no CRL-3216). The medium containing lentivirus was collected, filtered with Millex-GV 33 mm PVDF filter (Millipore SLGV033RS) and then concentrated with Amicon Ultra-15 centrifugal filters (Millipore UFC910024). Melanoma cells and normal human melanocytes were infected with the lentiviruses plus 10 µg polybrene, medium was changed the following day, and the cells were then incubated with puromycin (2.5 µg/ml) for 5 days. Cells were collected and processed for Western blotting with antibodies to target proteins as described [8]. Briefly, protein lysates (20 µg) were resolved on either 3–8% or 4-12% tris-acetate gel, transferred to membranes (NuPAGE Life Technologies, catalog no. NP0006), blocked in 5% milk, and probed with the primary antibodies using the concentrations recommended by the supplier (Supplementary Table S7).

The pLKO.1 shRNA treated cells were seeded in 96-well plates in triplicates 2 days after infection and tested for cell viability in the absence and presence of puromycin for 72 h with the CellTiter-Glo® Luminescent Cell Viability Assay (Promega Corporation, Madison, WI) as described [8]. Standard Error (SE) was calculated employing GraphPad Prism 7 software. The rate of apoptosis was measured using the Caspase-Glo® 3/7 Assay system (Promega catalog no. G8091) in a 96-well plate format following the manufacturer’s protocol.

Cell proliferation assays were performed with the VCPIP1 deubiquitinase inhibitors CAS-12290-201 and WH-9943 (both from Millipore/Sigma, St. Louis, MO Millipore/Sigma, St. Louis, MO), and the USP7/USP47 deubiquitinase inhibitor P-50429 (Probe Chem, Shanghai, P.R. China).

### RNA extraction and gene expression

We applied bulk RNA sequencing to determine differential gene expression in response to LZTR1 knockdown employing shRNA SCH002 as negative control [8]. RNAs from melanoma cells (YUSIK and YUSEEP) were extracted with Direct-zol™ RNA MiniPrep w/Zymo-Spin™ IIC Columns (Zymo Research catalog no. ZR2072) and 1 µg/sample were processed and sequenced by Yale Center for Genome Analysis.

### Generation of miniTurbo expression constructs

MiniTurbo coding sequence was amplified from 3xHA-miniTurbo-NLS_pCDNA3 (Addgene, catalog no 107172) using the forward 5′-TATCTCGAGTGCTAGCATCCCGCTGCTGAAC-3′ and reverse 5′-GTATTCGAACTGCAGCTTTTCGGCAGACCG-3′ primers that carry XhoI and BstBI restriction sites, respectively. The amplicon was then ligated into the Gateway™ expression vector pInducer20 (Addgene, catalog no 44012). The miniTurbo was sequenced and verified to be void of any mutation and the vector was named pIND20-ccdB-miniTurbo-HA. The entry clones pENTR223-LZTR1 and pDONR221_EGFP (Gateway™ pDONR™221 Vector GFP, catalog no 58250) were shuttled into the pIND20-ccdB-miniTurbo-HA using LR Clonase II (Thermo Fisher Scientific, catalog no. 11791020) following manufacturer’s protocol. The resulting pIND20-GFP-miniTurbo-HA and pIND20-LZTR1-miniTurbo-HA expression vectors were used in the BioID experiments.

### Proximity biotinylation, co-immunoprecipitation, Western blotting and proteomics

We used the doxycycline-inducible LZTR1-mini-TurboID-HA lentiviral vector (i.e., LZTR1 fused to biotin ligase tagged with HA) and GFP-TurboID as a control, for biotinylation of proximal proteins in cells [17]. Melanoma cells were infected with each plasmid, selected with puromycin, incubated with doxycycline (200 ng/ml) for 2–3 days followed by biotin (50 µM) for 24 h. Cells were harvested by scrapping on ice, centrifuged, washed with PBS, and lysed with buffer containing 50 nM TRIS-HCL, 150 mM NaCl, 10% Glycerol, 1% NP-40, 1 mM EDTA supplemented with phosphatase inhibitor (Thermo Fisher Scientific catalog no. 78428) and protease inhibitor (Thermo Fisher Scientific catalog no. 78425). Biotinylated proteins were captured with streptavidin magnetic beads (Pierce, catalog no. 88816) overnight, the beads were washed, and proteins were eluted with SDS sample buffer (35–50 µl) according to the manual’s procedure. Each experiment was performed in biological triplicate.

For co-immunoprecipitation, lysates from LZTR1-HA infected YUHIMO melanoma cells grown in the presence or absence of doxycycline for 3 days, were subjected to immunoprecipitation with anti-HA monoclonal antibodies magnetic beads (Pierce, Product No. 88836), overnight, in the cold. The beads were washed with Tris-buffered saline (TBS, Product No. 28379) containing 0.1% Tween™-20 Detergent, proteins were eluted with HA-peptide (MedChemExpress, HY-P0239) and subjected to mass-spectrometry. In addition, after co-immunoprecipitation, proteins were eluted with SDS sample buffer (35–50 µl) and subjected to Western blotting with antibodies to target proteins listed in Supplementary Table 6. The secondary antibodies used for detection were anti-rabbit IgG HRP linked (Cell signaling, cat number 7074S), anti-mouse IgG HRP linked (Cell signaling, cat number 7076S), HRP-conjugated Affinipure rabbit anti-goat IgG (H + L) (Proteintech cat number SA00001-4).

Biotinylated proteins were subjected to in-gel trypsin digestion exactly as previously described [18]. Briefly, each sample was run on a lane of an SDS-PAGE gel, the lane was excised and digested with trypsin for 16 h at 37 °C. The tryptic peptides were extracted from the gel and dried in a SpeedVac, followed by cleanup with ethyl acetate extraction. De-salting was performed with a C18 spin column (Thermo Fisher Scientific catalog number 89870), followed by resuspension in 35 µl of 0.1% formic acid (FA) and removal of particulates by centrifugation for 30 min at 15,000 rcf, 4 °C in an Eppendorf rotor. Liquid chromatography-mass spectrometry (LC-MS/MS) was performed in-house on an Easy-nLC 1200 (Thermo Fisher Scientific) in-line with a Thermo Fisher Scientific Q Exactive Plus Hybrid Quadrupole-Orbitrap mass spectrometer as described [18].

The proteomic data were analyzed using MaxQuant (version 2.0.3.0). Oxidation of methionine and protein N-terminal acetylation were set as variable modifications. Carbamidomethyl of cystine was set as fix modification. The data were searched against the human UniProt protein database (version 2021). For all analyses, a mass deviation of 20 ppm was set for MS1 peaks, MS/MS tolerance was 0.6 Da, and a maximum of two missed cleavages were permitted. Maximum false discovery rates were set to 1% both on peptide and protein levels. The minimum required peptide length was five amino acids. Protein quantitation was calculated by the label-free quantification and setting the label-free quantitation (LFQ) minimum ratio count to 2. Data normalization, filtering, and imputation were performed using Perseus (version 2.0.3.0). Missing values were imputed from a normal distribution with a downshift of 1.8 and a width of 0.15. *P* values (Student’s *t*-test, two-tailed) were calculated for experimental versus control using Perseus.

### Proteasome activity

YUZEST melanoma cells were infected with pLKO.1 shRNA shLZTR1 or Non-Mammalian shRNA SCH002 pLKO.1 vector (shControl) for 6 days and selected with puromycin. The cells were incubated with BioTrackerTM TAS2 Proteasome Activity Live Cell Probe (1:1000), a substrate probe that monitors the protease-like activity of the catalytic 20S proteasome (Sigma Aldrich SCT235), for 90 min in the dark, washed with PBS, trypsinized, and resuspended in flow cytometry sort buffer (PBS with 0.5% FBS) (following manufacturer’s protocol). Samples were analyzed on a BD LSRFortessa™ Cell Analyzer using the Blue488 laser with 515/20 filter and a voltage setting of 500. Data were analyzed using FlowJo v10 and Prism v10. The experiment was performed in triplicates to determine the rate of hydrolysis of TAS2 in LZTR1 knockdown and shControl melanoma cells.

### Mechanical compression

Mechanical compression was applied to cells using a method adapted from a previous study [19]. Briefly, 200,000 cells were seeded onto cellQART cell culture inserts (8 μm pore with TRAKETCH® Membranes, Sterlitech, Auburn, WA, SKU: 9308012) within 6-well plates (Corning, NY). Circular agarose gel cushions, 19 mm in diameter, were cut from sterile 0.7% (w/v) agarose sheets. These sheets were prepared by dissolving agarose (Sigma, A9539) in boiling PBS and pouring the mixture into 100-mm petri dishes. To apply compression, a gel cushion was gently placed on top of the cells in the cell culture inserts. A sterile flat-bottom cap from a 15 ml conical tube (weight: ~1 g) with additional varied weights was then gently placed on the agarose cushion. Depending on the weights used, 4.3 g or 7 g, this setup generated mechanical pressure on the cells of 111 Pa or 180.6 Pa, respectively. Inserts without any weight were used as control (0 weight). Cell culture media (±200 ng/ml doxycycline) was added to both sides of the transwells, and the cells were allowed to grow and invade for 5–6 days. Invading cells were counted with Invitrogen Countess II 2 FL Automated Cell Counter AMQAF1000 Thermo Fisher.

### Statistical analysis

All statistical analysis was performed using Prism version 10.0.0 for (GraphPad Software, Boston, Massachusetts USA). For single comparisons, unpaired two-sided *t*-test was used. For multiple comparisons, ordinary one-way ANOVA was used with correction for multiple hypothesis testing using the default method.

## Results

### Proximal LZTR1 proteins are enriched for ubiquitin proteasome pathway

**Fig. 1:**
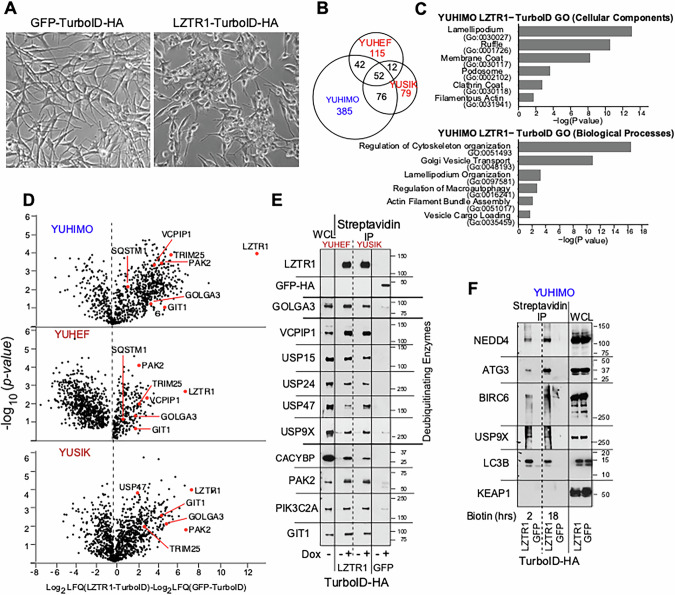
LZTR1-associated proteins in melanoma cells identified with proximity biotinylation. **A** Normal human melanocytes expressing GFP-TurboID-HA or LZTR1-TurboID-HA, as indicated. **B**–**D** Proteomic analysis of biotinylated proteins (see also Supplementary Table S2). LZTR1-TurboID or GFP-TurboID (control) were expressed in YUHIMO, YUHEF and YUSIK melanoma cells, followed by biotinylation, streptavidin enrichment and quantitative proteomics. **B** Venn diagram indicating the number of biotinylated proteins enriched (*p* < 0.05) over control in each cell line. Three biological replicates were analyzed for each cell line tested. **C** Selected Gene Ontology (GO) terms (Cellular Components and Biological Processes) enriched in YUHIMO LZTR1-TurboID proteomics data. For complete Gene Ontology analysis see Supplementary Table S2. **D** Volcano plots for enrichments (difference log_2_ LFQ, label-free quantitation value) versus significance (*p* value, Student’s *t* test) for experimental compared to control in each melanoma cell line tested. X and y axes for all three plots are on the same scale. Examples of proteins enriched in all three cell lines (LZTR1, GIT1, GOLGA3, PAK2, TRIM24), or in 1–2 cell lines (USP47, VCPIP1/VCIP135, SQSTM1) are highlighted in red. **E**, **F** Western blots validating LZTR1 streptavidin-proximal proteins. Melanoma cells expressing LZTR1-TurboID or GFP-TurboID in the absence or presence of doxycycline to induce expresion were incubated with biotin for 24 h (**E**) or for 2 and 18 h (**F**), and bead-bound proteins (IP), as well as whole cell lysates (WCL) were separated by SDS-PAGE and the membrane were probed with antibodies to the indicated proteins. Cells from acral or sun-exposed melanoma are marked blue and red, respectively.

We determined the proteins associated with LZTR1 in melanoma cells (YUHIMO, YUHEF and YUSIK) by employing proximity biotinylation (TurboID) and co-immunoprecipitation combined with LC-MS/MS proteomics [20, 21]. The mini-TurboID infected melanoma cells (YUHIMO) were incubated with biotin to generate a comprehensive map of cellular proteins that encounter LZTR1 during their trafficking or function for 2 h or 18 h, recognizing that the latter cannot distinguish between direct or indirect LZTR1 interactions. The cells were then harvested, and protein biotinylation in cell extracts was validated by blotting with Streptavidin-HRP conjugate (Supplementary Fig. S1A). In addition, 18-h biotinylated proteins were extracted from the gel and subjected to mass spectrometry with LFQ as described [22]. The mini-TurboID tag did not interfere with LZTR1 function, because the fusion protein conferred the malignant phenotype described before for LZTR1, i.e., induction of anchorage-independent growth, and formation of three-dimensional clusters as reported before [8] (Fig. 1A, compare GFP to LZTR1).

We identified hundreds of proteins enriched by LZTR1-TurboID over GFP-TurboID control (*p* < 0.05), with 52 proteins common to all three cell lines (Fig. 1B). Gene Ontology (GO) (Pantherdb.org) revealed LZTR1-proximal proteins related to the Golgi, autophagy, lamellipodium and actin filaments (Fig. 1C, D, Supplementary Table S2). The association was specific because canonical LZTR1 partners were present and validated as well; these include ubiquitin ligases [2], deubiquitinase, and proteins of the Golgi apparatus [1] such as NEDD4 (E3 ubiquitin ligase), ATG3 (E2 ubiquitin-like conjugating enzyme), BIRC6 (ubiquitin conjugating enzyme), LC3B (microtubule associated protein 1 light chain 3 beta MAP1LC3B), GOLGA3 (golgin A3), and the autophagy receptor SQSTM1 [5, 23–25] (Fig. 1D-F, and Supplementary Table S2). We confirmed the association with USP9X, and identified other deubiquitinases (DUBs) that interacts with LZTR1, such as VCPIP1, enriched in two cell lines; the Ubiquitin-Specific Proteases USP15, USP24, USP47, enriched in one or two cell lines; and, detected by Western blotting but not proteomics (Fig. 1E, F). In contrast, LZTR1 did not associate with Kelch-like ECH-associated protein (KEAP1) (Fig. 1F), which also acts as an orthogonal substrate adaptor protein for the E3 ubiquitin ligase complex, validating the specific association of LZTR1 with the indicated proteins.

We also recapitulated LZTR1 association with proteins that function in vesicle trafficking, adhesion, cytoskeletal organization described before, such as and survival such as CACYBP (calcyclin binding protein) [26], the p21 (RAC1) activated kinase 2 PAK2 [27], phosphatidylinositol-4-phosphate 3-kinase catalytic subunit type 2 alpha (PIK3C2A) [28, 29], and ARF-GTPase-activating protein GIT1 (GIT1) [30, 31] (Fig. 1E). However, we did not detect enrichment for RAS in melanoma cells (Supplementary Table S2), suggesting that LZTR1 is engaged in constitutive and tissue-specific interactions depending on the cell type. In addition, the results challenge the current notion that LZTR1 serves only as an adaptor protein in the ubiquitination process [2], and they are consistent with a model in which LZTR1 is in the Golgi apparatus in proximity to a diverse suite of ubiquitinases and DUBs, as well as other proteins, and it is a hub for bidirectional regulation of cell-type specific protein targets.

**Fig. 2:**
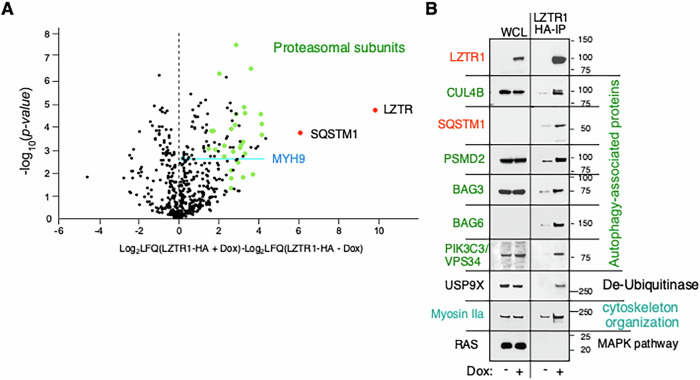
LZTR1 association with the proteosome. **A** Volcano plot showing enrichments of LZTR1 co-immunoprecipitated proteins (difference log_2_ LFQ, label-free quantitation value). **B** Western blot analysis validating LZTR1 assciated proteins involved in ubiquitin-protein ligase, proteasome and autophagosomes (CUL4B, SQSTM1, PSMD2, BAG3 and BAG6, and PIK3C3/VPS34), protein stablization (USP9X) and actin cytoskeleton organization (Myosin IIa) composed of MYH9, MYH10, MYH14. The top panel shows precipitated LZTR1-HA. The RAS panel confirmed lack of association with LZTR1. Non-induced YUHIMO cells were used as control.

To further identify associated proteins, we immunoprecipitated LZTR1-HA (described in reference [8]) from YUHIMO acral melanoma cells grown in the presence or absence of doxycycline (200 ng/ml) for 3 days using bead-bound anti-HA monoclonal antibodies. The eluted proteins were identified by liquid chromatography-mass spectrometry, and the results were compared to immunoprecipitated proteins from uninduced cells as a negative control. The data confirmed the physical association of LZTR1 with proteasomal and ubiquitin complexes such as PSMD2 (proteasome 26S subunit ubiquitin receptor, non-ATPase 2), CUL4B, SQSTM1, BAT3 complex (cochaperones BAG3 and BAG6) and phosphatidylinositol 3-kinase catalytic subunit type 3 (PIK3C3/VPS34) (Fig. 2A, B, Supplementary Table S3), but not with CUL3 (Supplementary Fig. S1B), substantiating the general role of LZTR1 in regulating protein stability in the secretory pathway as seen in HEK293 cells [5, 23]. The BAT3 complex supports pro-survival proteins that represents a therapeutic target in cancer cells [32], and the PI3K complex regulates autophagy [33, 34]. The co-immunoprecipitated proteins included actin-related proteins and actin cytoskeleton organization that we confirmed by the presence of myosin IIa (MYH9) (Fig. 2A, B, Supplementary Table S3). In contrast, none of the RAS family members were present in the mass spectrometry analysis of the BioID or LZTR1-HA pulled down proteins from melanoma cells (Supplementary Tables 2 and 3), a negative result that we rigorously confirmed by Western blotting (Fig. 2B).

### Proteins in the ubiquitination pathways maintains cell viability

We postulated that the proteins associated with LZTR1 have a role in melanoma cell survival and proliferation. We therefore tested the impact of depleting the E3 ubiquitin protein ligase NEDD4 because it is abnormally expressed in multiple cancers including melanoma and it is considered a good therapeutic target [35–37]. Indeed, our data show that downregulating NEDD4 in three independent melanoma cell lines (acral and sun exposed) induced growth arrest (Supplementary Fig. S2), supporting the notion that NEDD4 can be a good target to suppress melanomas.

We went on to explore the effect of DUBs on melanoma cell proliferation and LZTR1 expression. While current shRNA that target VCPIP1 (Valosin Containing Protein Interacting Protein 1) did not eliminate the target protein (Supplementary Fig. S3A), the VCPIP1 inhibitors CAS-12290-201 or WH-9943 had only a minimal effect on melanoma cell proliferation (Supplementary Fig. S3B–F), suggesting that the LZTR1-VCPIP1 interaction is not critical for melanoma cell viability. Downregulating USP15 or USP24 (ubiquitin specific peptidase 15 or 24) in YUHIMO acral melanoma cells with specific shRNAs had no effect on cell proliferation (Supplementary Fig. S4A, B), but knocking down USP47 induced growth arrest (Supplementary Fig. S4C). However, LZTR1 and USP47 did not co-immunoprecipitate (Supplementary Fig. S4D, HA IP), and downregulation of USP47 did not affect LZTR1 expression (Supplementary Fig. S4D), suggesting that their association is transient. Growth arrest in response to suppression of USP47 is likely due to its function in DNA damage and other apoptotic process [38].

**Fig. 3:**
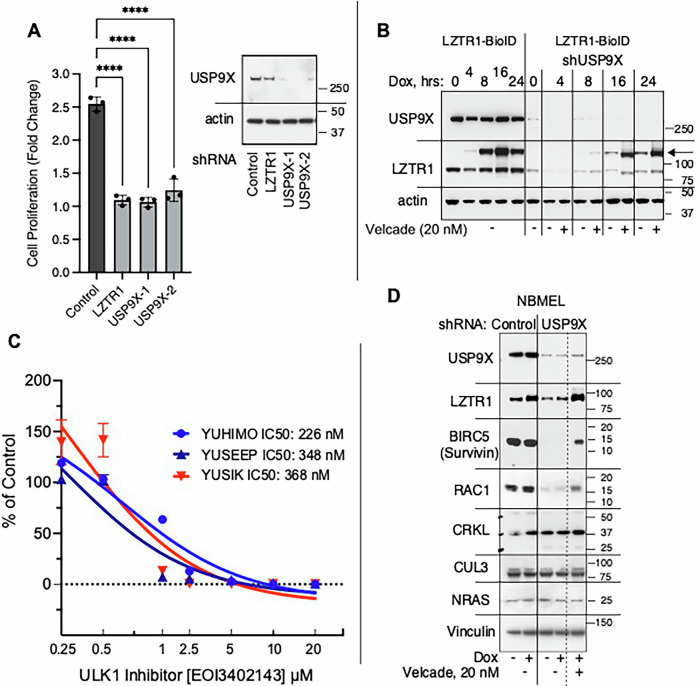
USP9X regulates LZTR1 stability. **A** USP9X shRNAs GAGAGTTTATTCACTGTCTTA and CGCCTGATTCTTCCAATGAAA (shUSP9X1 and shUSP9X2, respectively), inhibited YUHIMO acral melanoma cell proliferation compared to shRNA SCH002 vector pLKO.1 as control (shControl). **** indicates *p* < 0.0001 for comparison of each treated group to control using one-way ANOVA with Dunnett correction for multiple hypothesis testing. **B** Downregulation of USP9X in YUHEF sun-exposed melanoma cells suppressed the expression of endogenous LZTR1, as well as inducible LZTR1-TurboID, that were recovered with velcade (bortezomib). Cells were collected before (0) and at different time points after incubation with doxycycline (Dox) as indicated, and cell lysates were probed for USP9X and LZTR1, using actin as a control. Arrow points at LZTR1-BioID protein. **C** Cell proliferation in response to the DUB inhibitor EOAI3402, also known as G9. YUHIMO cells were the most sensitive to the drug (IC50 226 nM). **D** Normal human melanocytes (NBMEL) were treated with shRNA SCH002 (Control) or with USP9X shRNA (USP9X) for 6 days; velcade was added 21 h before harvest (+) to rescue proteins from degradation. The Western blot of cell lysates revealed downregulation of LZTR1, BIRC5, and RAC1 in response to USP9X knockdown that were recovered from degradation by velcade (+), validating the protecting role of USP9X from degradation of critical selected proteins. In contrast, there were no changes in the expression of CRKL, CUL3, NRAS and vinculin.

Of particular interest was USP9X because it interacts with LZTR1 in melanoma cells employing two independent approaches (Fig. 1F, G; Fig. 2B), and it was reported to be in the LZTR1-CUL3 complex [5, 23]. Downregulation of USP9X in YUSIK sun-exposed melanoma cells with two independent shRNA suppressed proliferation to the same extent as downregulation of LZTR1 (Fig. 3A). Furthermore, USP9X protects LZTR1 from ubiquitination and degradation because knocking down the protein suppressed the expression of endogenous LZTR1 as well as ectopically expressed LZTR1-TurboID, both recovered in the presence of the proteasome inhibitor velcade (bortezomib) (Fig. 3B). In support of this notion, EOAI3402143, an inhibitor of USP9X, USP24 and USP5 also suppressed the proliferation of three independent melanoma cell lines (Fig. 3C), probably by inducing apoptosis [39]. In contrast, under expression or overexpression of LZTR1 did not cause changes in the levels of USP9X (Fig. 3A, B, respectively). We also tested the effect of USP9X knockdown in normal human melanocytes (NBMEL) expressing LZTR1-HA and showed that USP9X maintained cell viability by protecting baculoviral inhibitor of apoptosis (IAP) repeat containing 5 (BIRC5) and Rac family small GTPase 1 (RAC1) from degradation (Fig. 3D). BIRC5 is an IAP that prevents apoptotic cell death, and RAC1 is a small GTPase that is activated by mutation in some melanomas and enhances melanoma cell growth even in the absence of mutation [40]. The effect of USP9X deletion on BIRC5 and RAC1 was specific because others, such as CRK like proto-oncogene, adaptor protein (CRKL), CUL3, and NRAS were not affected (Fig. 3D). We concluded that the deubiquitinase USP9X protects the stability of LZTR1 in melanoma and thus may itself represent a therapeutic target.

### Depletion of LZTR1 suppresses proteasome activity and induces apoptosis

**Fig. 4:**
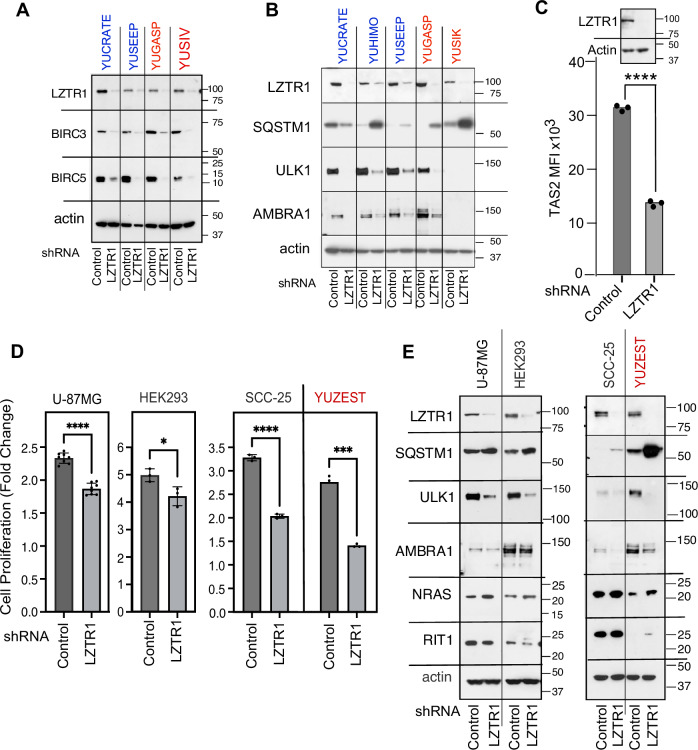
LZTR1 regulates apoptosis and autophagy. **A**, **B** Western blots showing changes in expression of BIRC3, BIRC5 (survivin), SQSTM1, ULK1 and AMBRA1 in response to depletion of LZTR1 (shLZTR1) compared to shRNA SCH002 (shControl) in melanoma cells from acral (blue) or sun-exposed (red) melanomas. **C** Histogram of proteasome activity in YUZEST-shLZTR1 (LZTR1), compared to YUZEST-SCH002 (Control), the background (no cells, probe only) was subtracted. MFI, mean fluorescent intensity. **** indicate significant change (*p* < 0.0001 by two-tailed *t*-test). Top shows downregulation of LZTR1 protein. **D** cell proliferation and **E** gene expression of non-melanocytic cell lines U-87MG, HEK293 and SCC-25 in response to LZTR1 knockdown compared to YUZEST melanoma cells. LZTR1 and control represent shLZTR1 and shRNA SCH002, respectively. **** indicates *p* < 0.0001, *** indicates *p* < 0.001, and * indicates *p* < 0.05 for comparison of each treated group to control using two-tailed *t*-test. The cell proliferation of the other melanoma cell lines was presented in Fig. 3 of reference [8].

Our proximity labeling and co-immunoprecipitation results suggested that LZTR1 regulates the stability and activity of proteins associated with autophagy, apoptosis and stress response in melanoma cells. We therefore downregulated LZTR1 with targeted shRNA and examined the impact on these pathways. We first showed that suppression of LZTR1 down-regulates BIRC3 and BIRC5 (known also as cIAP1 and cIAP2, respectively), a family of proteins that inhibit apoptosis (Fig. 4A). We then examined SQSTM1, because it associates with LZTR1-BioID and LZTR1-HA (Figs. 1, 2, Supplementary Tables 2 and 3), and show that it is upregulated in response to LZTR1 knockdown compared to shControl (SCH002) (Fig. 4B). SQSTM1 is phosphorylated by the serine/threonine-protein kinase ULK1 (autophagy-related kinase, Unc-51 like kinase-1, also known as ATG1) at its ubiquitin-association domain that regulates its binding to ubiquitinated proteins [12, 41]. Indeed, probing for ULK1 showed that the protein is downregulated in response to LZTR1 knockdown (Fig. 4B). The changes in protein levels were probably due to posttranslational modification and degradation because mRNA levels remain the same (Supplementary Table S4A, B). In addition, USP20, a ubiquitin-specific protease reported to stabilize ULK1 protein [42] was present at equal levels. Downregulating LZTR1 with another shRNA that targets a different site (nucleotides 2472-2492, NM_006767.4) had the same effect on ULK1 and SQSTM1 (Supplementary Fig. S4E).

ULK1 is a binding partner and functional interactor with AMBRA1 (Activating Molecule in BECN1-Regulated Autophagy protein 1), and both constitute the core of the autophagy machinery [43]. ULK1 phosphorylates AMBRA1, and AMBRA1 promotes Lys63-linked ubiquitination of ULK1 [43, 44]. AMBRA1 is of particular interest because it is part of Cul4-RING E3 ubiquitin ligase complex [43], and we identified CUL4 as an LZTR1 partner (Fig. 2B, Supplementary Table S2). The results show that, as is the case with ULK1, LZTR1 knockdown reduced the levels of AMBRA1 in acral and sun-exposed melanoma cells (Fig. 4B). Downregulation of ULK1 also suppressed melanoma cell proliferation, but this effect was not mediated by changes in AMBRA1 (Supplementary Fig. S5A, B), suggesting that induction of apoptosis in response to LZTR1 knockdown is mediated mostly by suppression of ULK1 that mediates the protective autophagy machinery. Interestingly, we could not detect ULK1 or AMBRA1 in untreated BRAF^V600E^ YUSIK melanoma cells (Fig. 4B), (the latter was reported to be tumor suppressor in BRAF-mutant melanoma [45]), suggesting that growth arrest (as reported in reference 8), is induced in these cells by another mechanism. Indeed, the cause of growth inhibition is likely due to an increase in MARK3 (microtubule affinity-regulating kinase 3) and a decrease in CDC25B (M-phase inducer phosphatase 2) (Supplementary Fig. S6). MARK3 is a serine/threonine-protein kinase activated by metabolic stress, that induces phosphorylation and degradation of CDC25B and CDC25C, leading to G2/M phase arrest [46]. We confirmed an increase in GTP-binding protein Rit1 (RIT1) levels, as reported before [47] (Supplementary Fig. S5B).

We further explored the impact of LZTR1 knockdown and downregulation of ULK1 on the proteasome (Fig. 4C). We measured the relative proteasome activity in LZTR1-depleted melanoma cells (LZTR1) compared to SCH002 infected cells (Control). The experiment was performed in triplicates using a cleavable activity-based bioluminescent probe that measures specifically the proteasome catalytic activity [48]. The results show that deletion of LZTR1 caused ~2.7-fold reduction in proteasomal activity (Fig. 4C, *p* < 0.0001 by two-tailed *t*-test). Since proteasome activity is required to remove misfolded proteins, it is possible that growth inhibition is in part mediated by the accumulation of damaged ubiquitinated proteins [49, 50].

We also tested if the changes in response to LZTR1 knockdown are unique for melanoma by applying the same procedure to other cell types, such as the glioblastoma U-87MG and the immortalized human embryonic kidney cells HEK293, used to study the LZTR1 effect on RAS [5, 23], as well as the unrelated squamous cell carcinoma SCC-25 cells. There was only a slight reduction in cell proliferation in U-87MG and HEK293 (~20%), associated with incomplete elimination of ULK1 and a minute effect on AMBRA1 and SQSTM1 (Fig. 4E). In contrast, there was 64% reduction in the rate of SCC-25 cells proliferation, levels similar to that of YUZEST melanoma cells (Fig. 4D), both displaying drastic loss of ULK1 and an increase in SQSTM1 (Fig. 4E). These results may explain the small effect of LZTR1 knockdown on U-87MG and HEK293 growth responses that set them apart from melanoma cells.

### LZTR1 provides growth advantage under stress and promotes cell migration and invasion

**Fig. 5:**
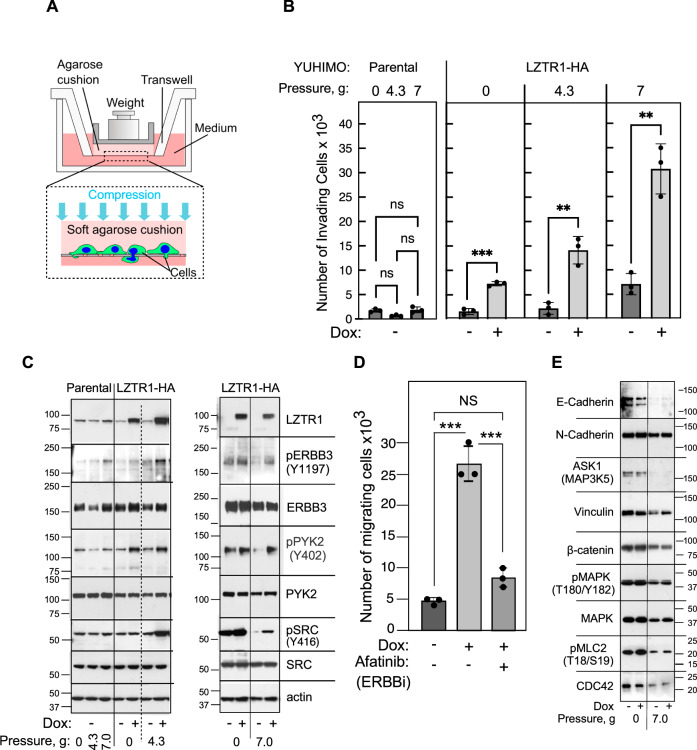
LZTR1 enhances cell invasion and migration under mechanical compression. **A** Schematic representation of the transwell piston adapted from Tse et al. [19]. **B** Histograms of YUHIMO-LZTR1-HA melanoma cell invasion in the absence or presence of mechanical stress and ±200 ng/ml doxycycline (Dox). Weights (4.3 g or 7.0 g) were added to half of the transwells (as indicated). Each condition represents average of triplicate wells. *** indicates *p* < 0.001, ** indicates *p* < 0.01 for comparison of each dox treated group to untreated using unpaired two-tailed *t*-test. ns not significant. **C** Western blot analysis of YUHIMO-LZTR1-HA cell extracts. The panels show LZTR1-HA induction, activation of ERBB3, PYK2 and SRC in response to mechanical stress. **D** Histogram of YUHIMO-LZTR1-HA melanoma cell invasion, ±100 ng/ml doxycycline (Dox) and ±the ERBB inhibitor afatinib (ERBBi, 0.5 µM) for 6 days. Values are average of triplicates transwell and *** indicates significant change (*p* < 0.001) by ANOVA test with Tukey’s correction for multiple comparisons. NS Not Significant. **E** Western blots showing that mechano-compressions downregulated some proteins that were not affected by LZTR1 expression. These blots were performed with the same samples used in (**C**) (left) and thus share actin levels.

Acral melanomas commonly arise in regions of physical or mechanical stress, such as the sole of the feet [51]. We therefor hypothesized that LZTR1 promotes melanoma cell survival under stress and tested its impact during mechanical compression, the most relevant types of stress for acral melanomas, as follows. YUHIMO acral melanoma cells expressing doxycycline inducible LZTR1-HA were seeded (200,000/well) on 6-well Cell Culture Inserts (8 µm pore size) in the absence or presence of doxycycline (100 nM). The following day, agarose cushions (0.7%) and conical tube caps containing pistons (final weight of 4.3 g or 7.0 g, corresponding to mechanical pressure of 111 Pa or 180.6 Pa, respectively) were added to some of the transwells (+pressure) using the setup adapted from Tse et al. [19] (Fig. 5A). Cell counts after 5 days incubation under stress, showed a significant increase in the number of YUHIMO-LZTR1-HA cells that invaded through the transwells and migrated to the bottom chamber in response to increased expression of LZTR1 (Fig. 5B). We probed the cell extracts for possible mediators of pressure-induced cell migration and identified activation of cell surface receptor ERBB3 (erb-b2 receptor tyrosine kinase 3, HER3) (Fig. 5C). Incubation with the ERBB inhibitor afatinib (0.5 µM, concentration that does not affect cell proliferation, Supplementary Fig. S7A), suppressed cell migration (Fig. 5D). Afatinib, also known as BIBW2992, binds to the active site of the kinase domain of ERBB family of tyrosine kinases, i.e., EGFR (Epidermal Growth Factor Receptor), ERBB2 (HER2), ERBB3 and ERBB4 and inhibits their activity [52, 53]. It is highly potent against EGFR and ERBB2 (IC_50_ values of 0.5 nM and 14 nM, respectively), compared to SRC, INSR (insulin receptor) HGFR (Hepatocyte Growth Factor Receptor) and VEGFR2 (Vascular endothelial growth factor receptor 2) (>4000, >100,000, 13,000 and >100,000 for respectively) [54]. Among the ERBB family of tyrosine kinases, ERBB2 and ERBB3 are expressed, whereas EGFR is sporadically expressed and ERBB4 is not expressed in melanomas ([55], and Supplementary Table S6 showing RNA expression in normal human melanocytes, nevi and 262 melanomas). ERBB3 itself has impaired tyrosine kinase activity and dimerizes with ERBB2 to become phosphorylated and acquire signaling potential. The two dimerize to function as an oncogenic unit [56]. Altogether, the results support the conclusion that LZTR1 mediates cell invasion under stress is in response to ERBB3 stimulation.

Further analysis revealed activation of two kinases known to be stimulated by ERBB-family of proteins and to associate with cell invasion, PYK2 (PTK2B, Protein-tyrosine kinase 2-beta) [11, 57] and SRC [8] (Fig. 5C, pPYK2 and pSRC). The effect of LZTR1 expression on ERBB3, PYK2 and SRC is notable, because other proteins involved in cell adhesion and cellular responses to changes in the microenvironment were downregulated under mechanical compression but were not affected by the increase in LZTR1 levels (Fig. 5E). In addition, the overexpression of LZTR1 did not change the levels of NRAS or RIT1 as reported before for RAS [2] and did not affect LC3B, an autophagosome marker [58] (Supplementary Fig. S7B).

## Discussion

We performed comprehensive studies to decipher the molecular pathway of LZTR1 oncogenic activity in melanomas compared to other cell types. Our work suggests that LZTR1 acts as a “safeguard” for melanoma cells. It protects the expression of two critical genes required for cell proliferation, ULK1 and AMBRA1, involved in proteasomal activity [41, 59–61] and induces invasion under mechanical stress *via* activation of ERBB3, PYK2 and SRC. Interestingly, in HEK293 and U-87MG cells routinely used to study RAS regulation in response to LZTR1 knockdown, ULK1 and AMBRA1 were only partially depleted, the likely basis for the differential response between these cells and melanomas. Altogether, LZTR1 belongs to the class of “double-agent” genes with tumor suppressive and oncogenic potential under different cellular contexts as described for *TP53* and *FAS* (Fas cell surface death receptor) as well as other genes [62].

ERBB3, SRC and PYK2 are known to have major roles in melanoma metastasis and drug resistance. ERBB3 is overexpressed in acral melanoma, and its elimination decreased anchorage-independent growth [63]. The stressed-induced ERBB3 activity described here is reminiscent of the matrix-dependent EGFR mechano-sensitization in squamous cell carcinoma invasion [64], and the induction of in vivo myeloid cell migration during adaptation to cardiac pressure overload [65]. Interestingly, a recent case report describes EGFR-mutated lung adenocarcinoma resistance to osimertnib due to the presence of novel LZTR1 germline mutation [66]. ERBB3 and SRC associate with melanoma cell migration [67, 68], and confer drug resistance to the BRAF inhibitor vemurafenib, that can be alleviated by the ERBB-inhibitor afatinib [57, 69–72]. Indeed, afatinib, is approved by the FDA as a third-line single agent therapy for head and neck squamous cell carcinoma.

PYK2 is a focal adhesion kinase that reorganizes the actin cytoskeleton, regulates proliferation, promotes cell migration and spreading [10, 11, 57, 73, 74]. Furthermore, active PYK2 localizes at focal adhesions and invadopodia in melanoma cells and is correlated with extracellular matrix degradation [11]. The dual FAK/PYK2 inhibitor PF-431396 inhibits both focal adhesion and invadopodia activities, thereby reducing both migration and ECM degradation [11].

Proteomic analyses of proximal biotinylated or co-immunoprecipitated proteins confirmed known LZTR1 interactors, such as several ubiquitin-specific proteases (USPs), 26S proteasome non-ATPase regulatory subunits (PSMDs), proteins responsible for ubiquitin-mediated degradation (the BAG3/6 complex) and DUBs. Of interest, the results showed that LZTR1 interacts with CUL4B but not with CUL3. This is important because downregulation of RAS family of proteins by LZTR1 in other cellular systems is mediated by interacting with CUL3 in complex with RING E3 ubiquitin ligase [4, 75]. As the RAS family of proteins are not affected by LZTR1 expression levels in the melanoma system, we speculate that the functional specificity of CUL3-E3 and CUL4B-E3 ubiquitin-protein ligase complexes may be different and may mediate the ubiquitination of different target proteins. Interestingly, elevated CUL4B expression in cutaneous melanomas is associated with reduced overall survival and poor prognosis [76], suggesting that it is needed to maintain the malignant phenotype, possibly via interaction with LZTR1.

The association with USP9X might have clinical significance. USP9X has multiple targets including survivin (BIRC5) and RAC1 [77, 78]. It is elevated in melanoma compared to normal skin or benign lesions and has synthetic lethality in combination with MEK inhibitors [79]. It has a role in several human cancers such as lymphoma, myeloma, ductal, colon, prostate, and small-cell lung adenocarcinomas. It promotes cell survival and is considered a potential therapeutic target [39, 77, 78, 80, 81]. Genetic modifications in USP9X are present in different cancer types, as listed in cBioPortal database for Cancer Genomics [82]; interestingly, 50% of the 36 mutations are in melanomas. Pharmacological approaches to suppress DUBs for therapeutic benefit are currently in development [83].

The role of LZTR1 in melanomas is not restricted to proteasomal functions but expands to include the actin cytoskeleton such as myosin IIa (Fig. 2C). Myosin IIa controls cellular migration and filipodia formation, and plays a role in cell shape, cell spreading, cell cycle, cytoskeleton reorganization, focal contacts and lamellipodial extension [84, 85]. It is tempting to speculate that this association participates in cell detachment and spheroid formation when LZTR1 is overexpressed in normal human melanocytes [8].

In conclusion, the unique activity of LZTR1 in melanomas renders it, and its associated proteins, good therapeutic targets. ULK1 inhibitor DCC-3116 is already in clinical trials and additional drugs are being developed [41]. The relevance of dysregulated E3 ubiquitin ligase to malignant transformation insighted clinical interest in developing inhibitors to these enzymes [86]. For example, two possible inhibitors were identified for NEDD4 [87]. In addition, the application of targeted protein degradation by PROTACs to the clinic may facilitate this goal [88, 89]. PROTAC degraders for the focal adhesion kinases (such as PYK2) are under development for cancer therapy [90].

## Supplementary information

Supplementary Information LZTR1 is a melanoma oncogene that promotes invasion and suppresses apoptosis

dataset S2

dataset S1, dataset S3, dataset S4A, dataset S4B, dataset S5, dataset S6, dataset S7

## Data Availability

MS-based proteomics data are available via PRIDE with accession number PXD054157. Project accession: PXD054157; Token: hid0JLIvxMh4.
