# Supplementary material for: LZTR1 is a melanoma oncogene that promotes invasion and suppresses apoptosis
Source: Oncogene. 2025 Aug 30;44(41):3974–84. doi: 10.1038/s41388-025-03538-2 (PMC12500468; doi:10.1038/s41388-025-03538-2)
Supplement: Supplementary file 1 — Supplementary Information LZTR1 is a melanoma oncogene that promotes invasion and suppresses apoptosis [file 41388_2025_3538_MOESM1_ESM.docx]

**Supplementary Information**

**LZTR1 is a melanoma oncogene that promotes invasion and suppresses apoptosis**

**Table of Contents**

**Supplementary Figures**

Supplementary Fig. S1: a, LZTR1-BioID interacting proteins; b, lack of CUL3 in LZTR1-HA co-immunoprecipitate.

Supplementary Fig. S2: Growth arrest in response to NEDD4 knockdown with shRNA. Supplementary Figure S3: VCPIP1 in melanomas.

Supplementary Fig. S4: Responses of YUHIMO acral melanoma cells to the deubiquitinases USP15, USP24 and USP47.

Supplementary Fig. S5. Melanoma cell response to ULK1 knockdown

Supplementary Fig. S6: Regulation of several proteins in response to LZTR1 knockdown**.**

Supplementary Fig. S7: A, Growth response of YUHIMO-LZTR1-HA to ERBB inhibitor afatinib.

B. Negative results in response to mechano-compressions

**Supplementary Tables**

Supplementary Table S1: Characteristics of melanoma cell lines.

Supplementary Table S2: TurboID data (independent excel file)

Supplementary Table S3: co-IP data

Supplementary Table S4A: YUSEEP Gene expression in response to shLZTR1 compared to control (CT).

Supplementary Table S4B: YUSIK Gene expression in response to shLZTR1 compared to control (CT)

Supplementary Table S5: Lentiviral vectors MISSION pLKO.1 puromycin bearing shRNA used to test the effect of gene-specific downregulation on cell proliferation.

Supplementary Table S6: Gene expression in normal human melanocytes, nevi and melanomas.

Supplementary Table S7: List of antibodies used in this study (independent excel file)

|  |  |
| --- | --- |
| **A**  **** | **B**  **** |

**Supplementary Fig. S1**: **A** **LZTR1-BioID interacting proteins.** Western blot of proteins extracted from three biotin-labelled melanoma cell lines detected with streptavidin-HRP. The melanoma cells were treated (+) or untreated (-) with doxycycline (Dox) for 2 days before harvest. **B, lack of CUL3 in LZTR1-HA immunoprecipitate**. Anti-CUL3 Western blot of LZTR1-immunneprecipitates (IP) and whole cell lysate (WCL) of melanoma cells, treated and untreated with doxycycline for 2 days before harvest.

| **** | **** |
| --- | --- |

**Supplementary Fig. S2: Growth arrest in response to NEDD4 knockdown with shRNA.** Downregulation of NEDD4 (shNEDD4) in three independent melanoma cell lines (right panel) induced growth arrest (left panel). **** indicates P-value < 0.0001 for comparison of each treated group to control using one-way ANOVA with Dunnett correction for multiple hypothesis testing. Blue (YUCRATE and YUHIMO) and brown (YUSIK) are melanoma from acral and sun exposed melanomas, respectively.

| **A**  **** | **B**  **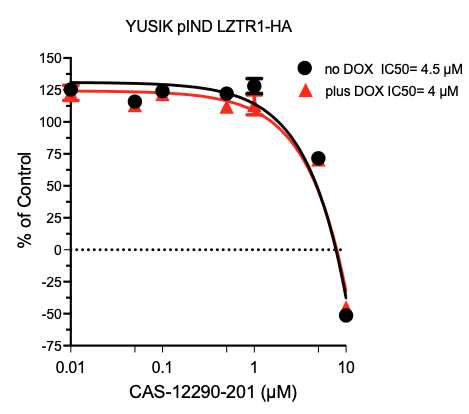** |
| --- | --- |
| **C**  **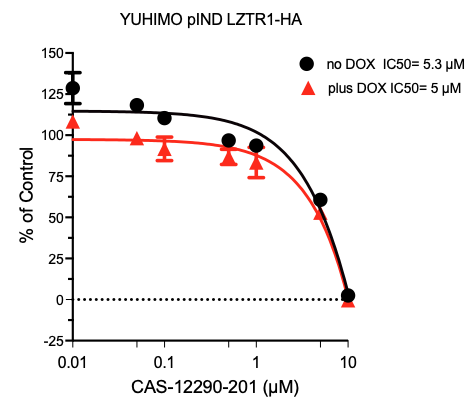** | **D 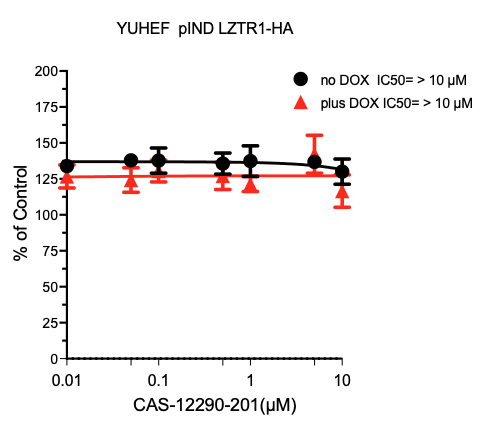** |
| **E**  **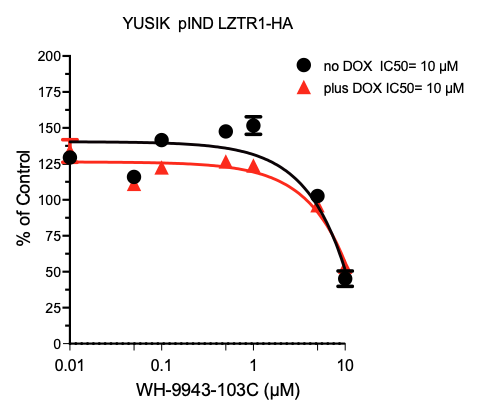** | **F**  **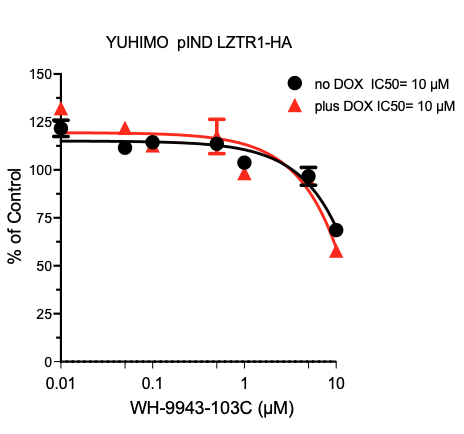** |

**Supplementary Fig. S3: VCPIP1 in melanomas**. **A** Western blot of YUHIMO cell extracts expressing LZTR1-HA in response to doxycycline (Dox), showing that the only available VCPIP1 shRNA (Supplementary Table 5) did not eliminate the target protein. **B-F** Cell proliferation in response to VCPIP1 inhibitors CAS-12290-201 or WH-9943.

| **A**  shUSP15   | **B**  shUSP24   | **C**  shUSP47  **** | **D**  **** |
| --- | --- | --- | --- |
| **** | **** | **** | **E**   |

**Supplementary Fig. S4. Responses of YUHIMO acral melanoma cells to the deubiquitinases USP15, USP24 and USP47**. **A-C** show cell proliferation (top, fold change compared to control), and protein expression (bottom) by Western blots related to shRNA knockdown of each deubiquitinase). Minus (-) indicates shControl (SCH002), and plus (+) the shRNA to the target protein. The shRNAs are described in Supplementary Table 5. **D** USP47 does not associate with LZTR1, as demonstrated by co-immunoprecipitation assay. **E** Effects on ULK1 and SQSTM1 recapitulated with shRNA targeting a different LZTR1 mRNA site [NM_006767.4](https://www.ncbi.nlm.nih.gov/nucleotide/NM_006767.4?report=genbank&log$=nuclalign&blast_rank=2&RID=9S1J8AW3013) (nucleotides 2472-2492), compared to shRNA targeting nucleotides 1162-1182 used in all other experiments and in panel b (listed in Supplementary Table 4). **** indicates P-value < 0.0001, *** indicates P-value < 0.001, and ** indicates P-value < 0.01 for comparison of each treated group to control using one-way ANOVA with Dunnett correction for multiple hypothesis testing. NS, Not Significant.

**Supplementary Fig. S5. Melanoma cell response to ULK1 knockdown. A** Downregulation of ULK1 suppresses melanoma cell proliferation. **** indicates P-value < 0.0001, *** indicates P-value

< 0.001 for comparison of each treated group to control using one-way unpaired t-test. **B** Downregulation of ULK1 does not affect the expression of AMBRA1 or LZTR1.

**Supplementary Fig. S6. Regulation of several proteins in response to LZTR1 knockdown.** LZTR1 knockdown regulates proteins associated with control of cell cycle**.** Western blots showing changes in expression of MARK3, CDC25B and RIT1, but not NRAS. The melanoma cells were derived from acral (blue), or sun-exposed (red) melanomas and the cell extracts are the same as presented in Figure 4B. Minus (-) and plus (+) indicate treatments with shControl (SCH002) or shLZTR1, respectively.

| **A**   | **B**  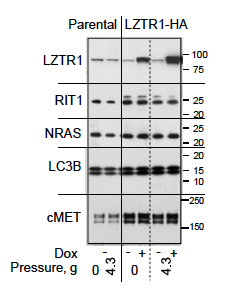 |
| --- | --- |

**Supplementary Fig S7: A Growth response of YUHIMO-LZTR1-HA to ERBBi afatinib.** YUHIMO-LZTR1-HA melanoma cells were grown in OptiMEM (Invitrogen, Carlsbad, CA) supplemented with 5% fetal calf serum and antibiotics in the absence and presence of doxycycline (100 ng/ml). Melanoma cells were seeded in 96-well plates in triplicate or quadruplet wells, with increasing concentrations of Afatinib for 72 hrs. Cell proliferation was measured with the CellTiter- Glo® Luminescent Cell Viability Assay (Promega Corporation, Madison, WI). The IC50 (the dose that elicits 50% inhibition compared to vehicle control) was calculated from the slope of the drug response by linear interpolation employing GraphPad Prism 7 software. **B Negative results in response to mechano-compressions.** Western blots showing a set of proteins not affected by mechano-compressions or LZTR1 expression. These blots were performed with the same samples used in Figure 5C and thus share actin levels.

**Supplementary Table 1**: Characteristics of melanoma and other cell lines.

| **Cell Line** | **Tumor Type** | **BRAF or NRAS Mutation** | | **Other Oncogene Mutations/changes** | | **References (WES)*** |
| --- | --- | --- | --- | --- | --- | --- |
| YUCRATE | Acral | | BRAF p.G469A | | MAPK6 p.E520K; MC1R p.R163Q. | 2 |
| YUHIMO | Acral | | PDE4DIP-BRAF fusion | | CDKN2A loss; CDKN2B loss; TERT Gain; LZTR1 Gain; CRKL Gain, EP300 Gain; SOX10 Gain; SRC Gain. | 2 |
| YUSEEP | Acral | | GOLGA4-RAF1 fusion | | EWSR1 p.G290E; CDKN2A loss; CDKN2B loss; TERT Gain; CCND1 Gain; LZTR1 Gain; CRKL Gain, EP300 Gain; SOX10 Gain. | 3 |
| YUSIK | Sun-exposed | | BRAF p.V600E | | MAP2K3 p.P162L; PTEN p.E288K. | 2 |
| YUHEF | Sun-exposed | | NRAS p.Q61L | | RAC1 p.P29S; NF1 p.Q853X; BAG3 p.P209L; PTPRD p.G203E | 2 |
| YUGASP | Sun-exposed | | NRAS p.Q61L | | NF1 LOH; CDKN2A-loss; FANCA p.P667L; MITF null; ATXN1 p.Q581H; RICTOR p.S1084L; p.WNK3 S1393L. | 3 |
| YUSIV | Sun-exposed | | PDE8A-RAF1 fusion | | NF1 p.L626F; BRCA1 p.V772A; PTEN p.E288K; TRRAP p.S722F; PCDHGA1 p.P155L. | 1 |
| YUZEST | Sun-exposed | | BRAF p.V600E | | CDKN2A p.R58X; SMARCA4 p.P1090S; BRCA1 p.R496C; MC1R p.R151C. | not published |
| Other cell lines | | | | | |  |
| SCC-25 | Squamous cell carcinoma | | ATCC, CRL-1628 (American Type Culture Collection) | | |  |
| HEK293 | Embryonic Kidney Cells | | ATCC (American Type Culture Collection ) | | |  |
| U87-MG | Glioblastoma | | ATCC (American Type Culture Collection ) | | |  |

**References**

1) Krauthammer, M., Kong, Y., Bacchiocchi, A., Evans, P., Pornputtapong, N., Wu, C., McCusker, J. P., Ma, S., Cheng, E., Straub, R., Serin, M., Bosenberg, M., Ariyan, S., Narayan, D., Sznol, M., Kluger, H. M., Mane, S., Schlessinger, J., Lifton, R. P., and Halaban, R. (2015) Exome sequencing identifies recurrent mutations in NF1 and RASopathy genes in sun-exposed melanomas. Nat Genet **47**, 996-1002

2) Krauthammer, M., Kong, Y., Ha, B. H., Evans, P., Bacchiocchi, A., McCusker, J. P., Cheng, E., Davis, M. J., Goh, G., Choi, M., Ariyan, S., Narayan, D., Dutton-Regester, K., Capatana, A., Holman, E. C., Bosenberg, M., Sznol, M., Kluger, H. M., Brash, D. E., Stern, D. F., Materin, M. A., Lo, R. S., Mane, S., Ma, S., Kidd, K. K., Hayward, N. K., Lifton, R. P., Schlessinger, J., Boggon, T. J., and Halaban, R. (2012) Exome sequencing identifies recurrent somatic RAC1 mutations in melanoma. Nat Genet **44**, 1006-1014

3) Farshidfar, F., Rhrissorrakrai, K., Levovitz, C., Peng, C., Knight, J., Bacchiocchi, A., Su, J., Yin, M., Sznol, M., Ariyan, S., Clune, J., Olino, K., Parida, L., Nikolaus, J., Zhang, M., Zhao, S., Wang, Y., Huang, G., Wan, M., Li, X., Cao, J., Yan, Q., Chen, X., Newman, A. M., Halaban, R. (2022) Author Correction: Integrative molecular and clinical profiling of acral melanoma links focal amplification of 22q11.21 to metastasis. Nat Commun **13**:898. https://doi.org/10.1038/s41467-022-28566-4

*Our Melanoma Exome Sequencing phs000933.v4.p1 is publicly available dbGaP: https://www.ncbi.nlm.nih.gov/projects/gap/cgi-bin/study.cgi?study_id=phs000933.v4.p1

as well as our RNA-Seq data:

[http://fcb.ycga.yale.edu:3010/nDe7bcV5Mmv9VBQVDaEUwHI_4pu2967/yale_rnaseq](https://nam12.safelinks.protection.outlook.com/?url=http%3A%2F%2Ffcb.ycga.yale.edu%3A3010%2FnDe7bcV5Mmv9VBQVDaEUwHI_4pu2967%2Fyale_rnaseq&data=05%7C02%7Cruth.halaban%40yale.edu%7C500600786f774a11470c08dcf20587e4%7Cdd8cbebb21394df8b4114e3e87abeb5c%7C0%7C0%7C638651352783145304%7CUnknown%7CTWFpbGZsb3d8eyJWIjoiMC4wLjAwMDAiLCJQIjoiV2luMzIiLCJBTiI6Ik1haWwiLCJXVCI6Mn0%3D%7C0%7C%7C%7C&sdata=5ht20EhuCzYIjfg9YvVEPVxj2xRWOsV2Pqqia26NTj4%3D&reserved=0)

**Supplementary Table 5: Lentiviral vectors MISSION pLKO.1 puromycin bearing shRNA used to test the effect of gene-specific downregulation on cell proliferation.**

|  |  |  |  |  |  |
| --- | --- | --- | --- | --- | --- |
| **Gene** | **Sigma Designation** | **Clone ID** | **Region** | **Sequence** |  |
|  |  |  |  |  |  |
|  |  |  |  |  |  |
| LZTR1 | TRCN0000181000 | NM_006767 | CDS | GATGTGTTTGGCCTGGACTTT (1162-1182)* |  |
| LZTR1 | TRCN0000180670 | NM_006767 | CDS | GCACATCATTGTGCACCAGTT (2472-2492)* |  |
| ULK1 | TRCN0000000835 | NM_003565 | 3’UTR | GCCCTTTGCGTTATATTGTAT |  |
| ULK1 | TRCN0000195477 | NM_003565 | CDS | CGCATGGACTTCGATGAGTTT |  |
| ULK1 | TRCN0000000838 | NM_003565 | CDS | ACATCGAGAACGTCACCAAGT |  |
| USP9X | TRCN0000007361 | NM_004652 | 3'UTR | GAGAGTTTATTCACTGTCTTA |  |
| USP9X | TRCN0000007364 | [NM_004652.3](https://www.ncbi.nlm.nih.gov/nuccore/NM_004652.3?report=genbank) | CDS | CGCCTGATTCTTCCAATGAAA |  |
| USP15 | TRCN0000231593 | NM_006313 | CDS | GCAGGTCCTTGCCGCTATAAT |  |
| USP15 | TRCN0000231592 | NM_006313 | CDS | GATACAGAGCACGTGATTATT |  |
| USP15 | TRCN0000007568 | NM_006313 | CDS | GCTCTTGAGAATGTGCCGATA |  |
| USP24 | TRCN0000245776 | NM_015306 | CDS | ACAATACTGTGACCGTATAAA |  |
| USP24 | TRCN0000245779 | NM_015306 | CDS | CTCTCGTATGTAACGTATTTG |  |
| USP47 | TRCN0000007695 | NM_017944 | CDS | GCAGCTTTCAAACAACATTTA |  |
| VCPIP1 | TRCN0000236144 | NM_025054 | CDS | GTGTGCCTCAGGACCTTATTA |  |

SHC002 MISSION® pLKO.1-puro non-mammalian shRNA Control Plasmid DNA was used as negative control.

*Based on NM_006767.4
